# Supplementary material for: A recently collected Xanthomonas translucens isolate encodes TAL effectors distinct from older, less virulent isolates
Source: Microb Genom. 2024 Jan 8;10(1):001177. doi: 10.1099/mgen.0.001177 (PMC10868612; doi:10.1099/mgen.0.001177)
Supplement: Supplementary material 1 [file mgen-10-1177-s001.pdf]

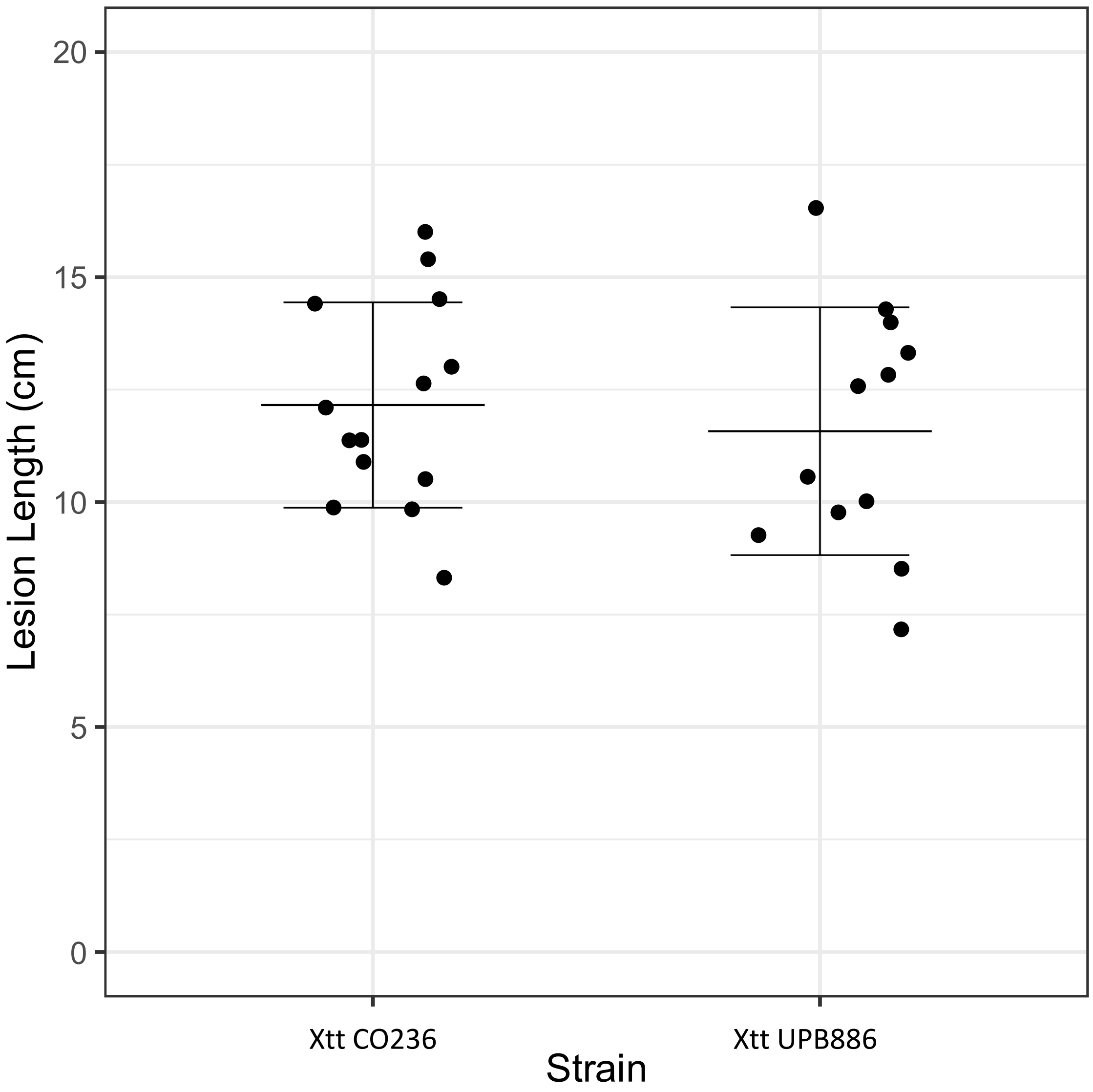

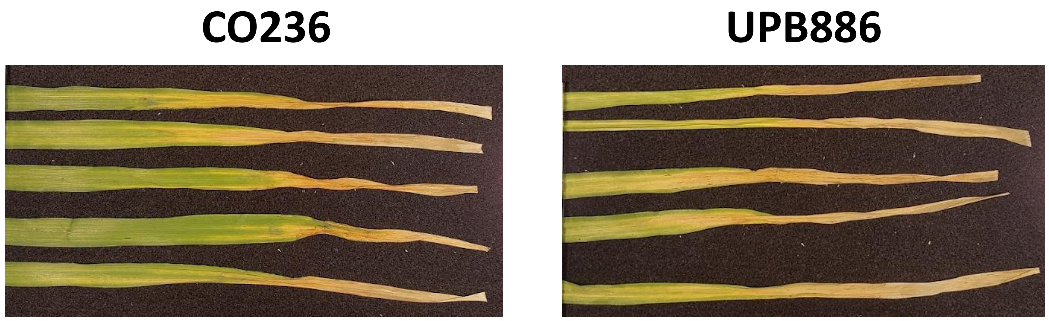

Supplemental Figure S1. Lesion lengths from clip inoculations of CO236 and UPB886 in barley (var. Morex) leaves. Lesions were measured 14 days post clip-inoculation. No significant difference was found between Colorado isolate and UPB886. Significance values determined by Wilcoxon test in R software (p value = 0.55, n=12).

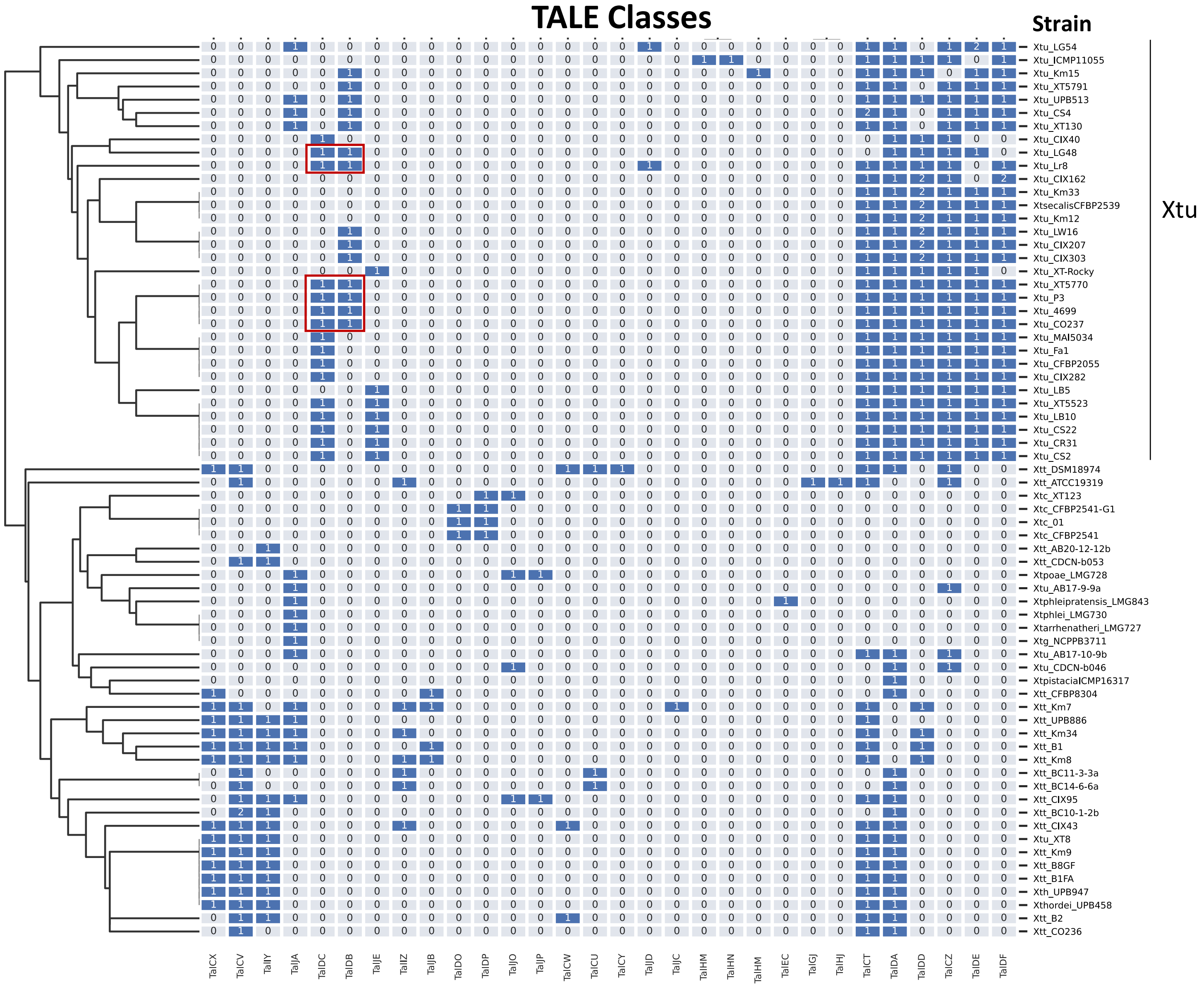

Supplemental Figure S2. Effector analysis of all *X. translucens* isolates. TALE classes in published *X. translucens* genomes. Numbers represent the number of copies of each TALE present in the genome. The TALE classes were annotated with AnnoTALE (Grau et. al, 2016).

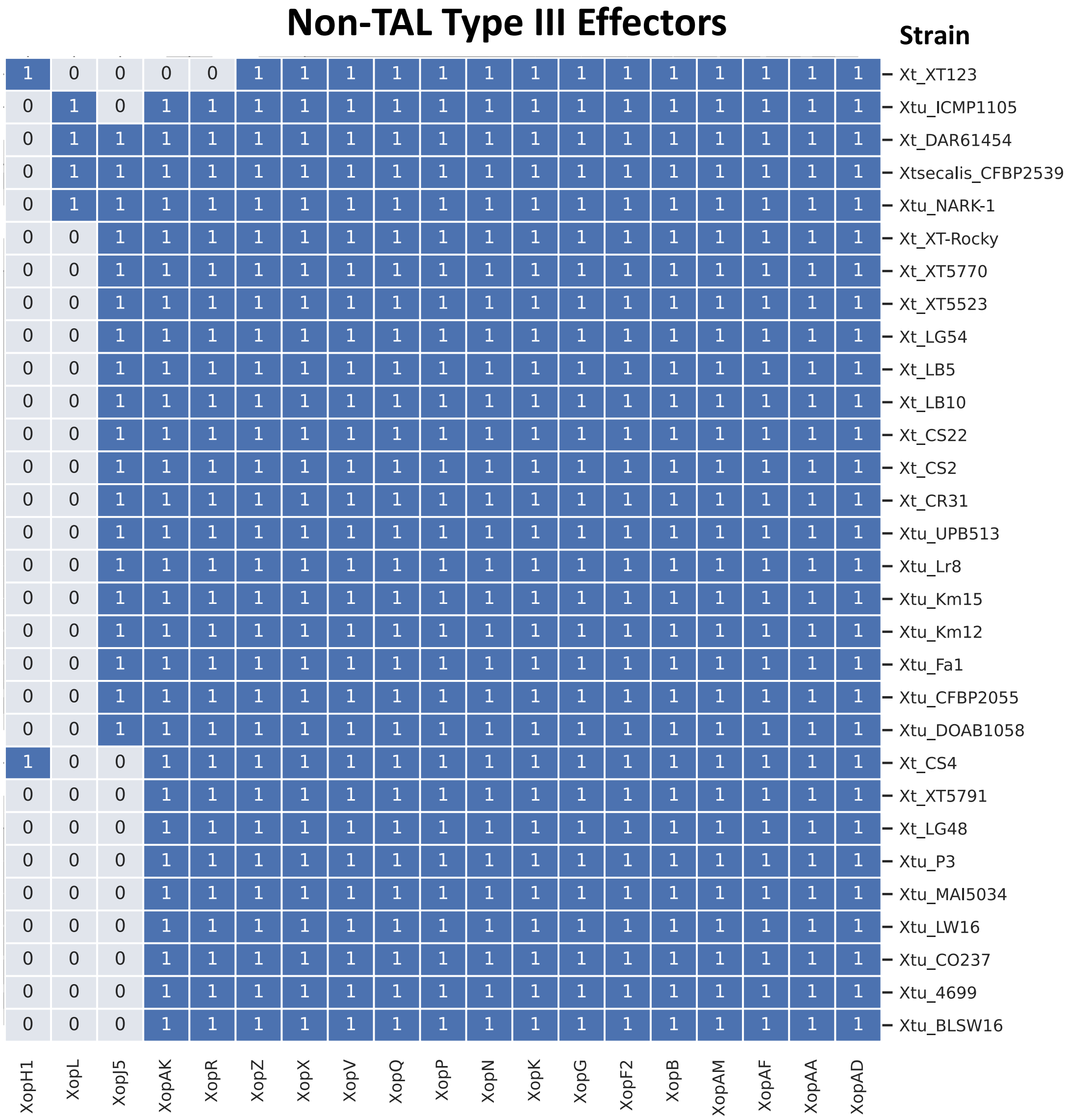

Supplemental Figure S3. Non-TAL Type III effector homologs were determined using known *Xanthomonas* effectors as the query (obtained from: http://www.biopred.net/xanthomonas/t3e.html) and conducting a BlastP search in published *X. translucens* genomes. Numbers represent the number of copies of each effector in the genome.

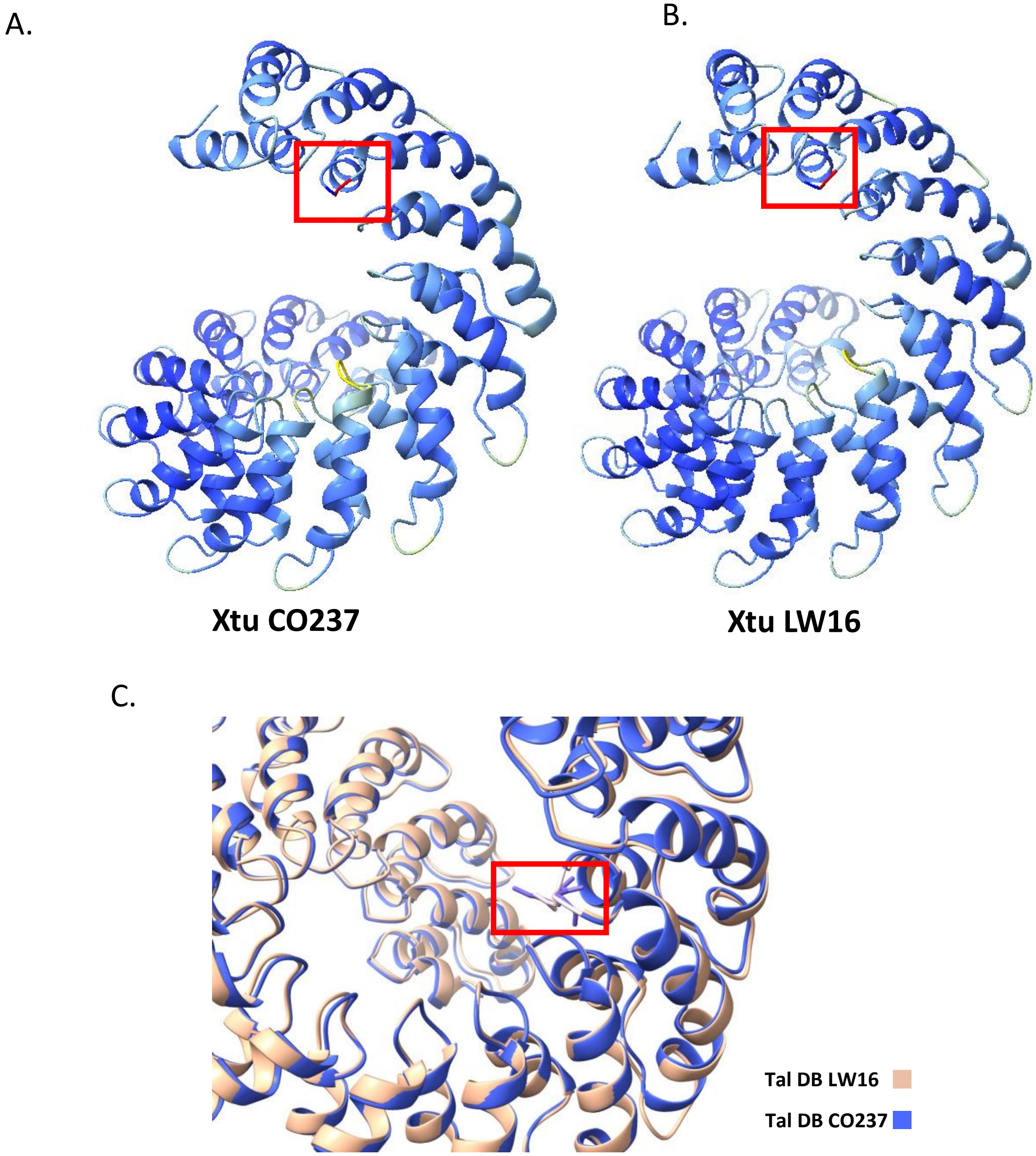

Supplemental Figure S4. Protein structure prediction of the Central Repeat Region (CRR) of TalDB in Xtu CO237 (A) and Xtu LW16 (B). Alphafold2 was used to predict the protein structure of the CRR of two TALEs. Red boxes highlight the mutation in the third RVD (NG -> KG) of the TalDB class of the low virulence strain LW16. **C.** The overlay of both protein structures shows no apparent difference of the folding between the CRRs.

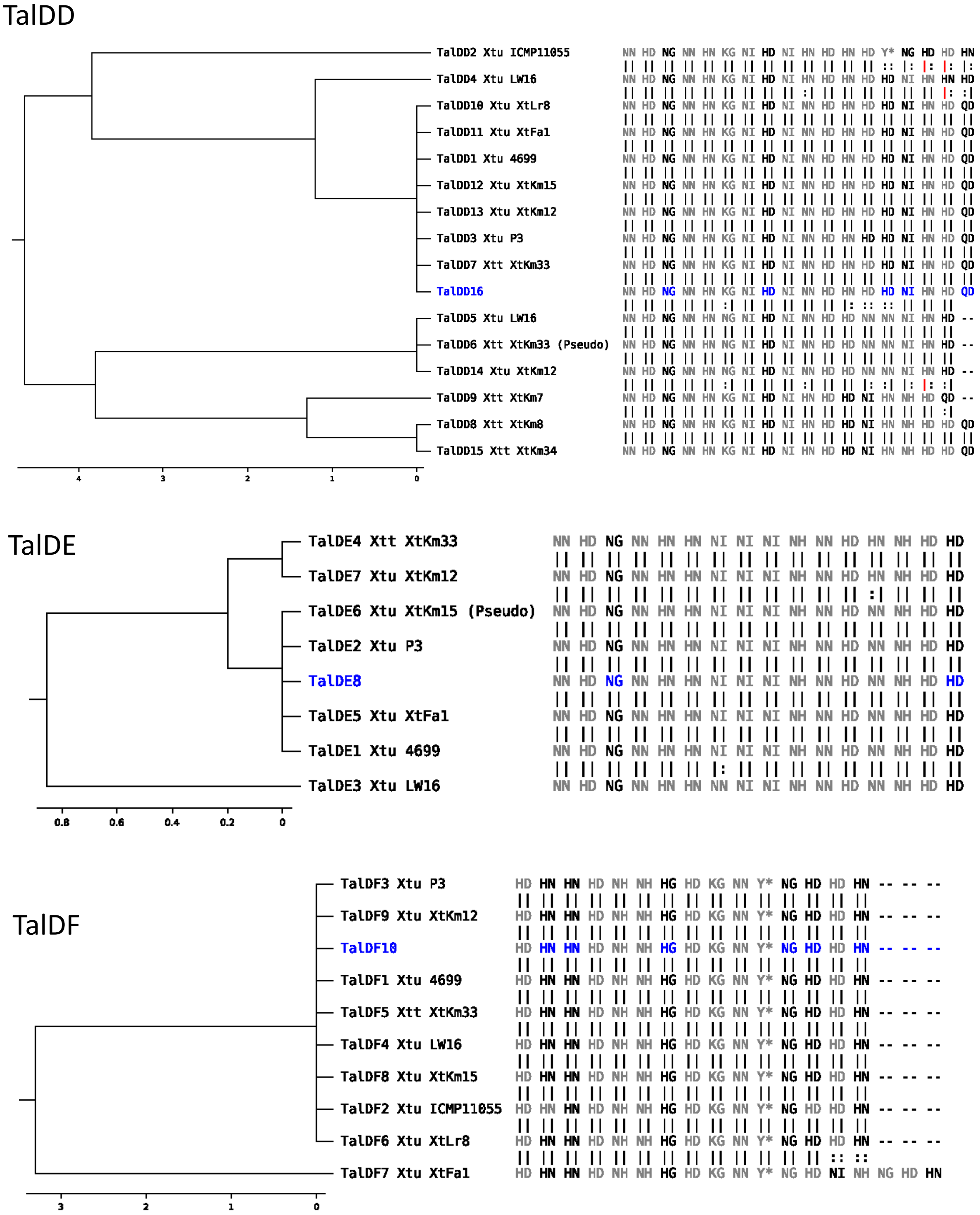

Supplemental Figure S5. TALE classes in *X. translucens* pv. undulosa. TALE classes assigned by AnnoTALE. Xtu CO237 TALEs are highlighted in blue in each tree class (continued on next page).

TalCZ

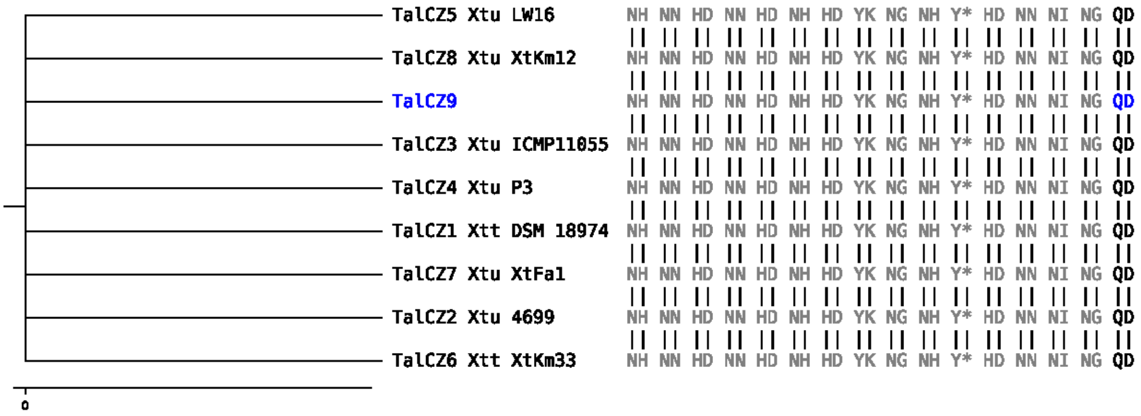

TalCT

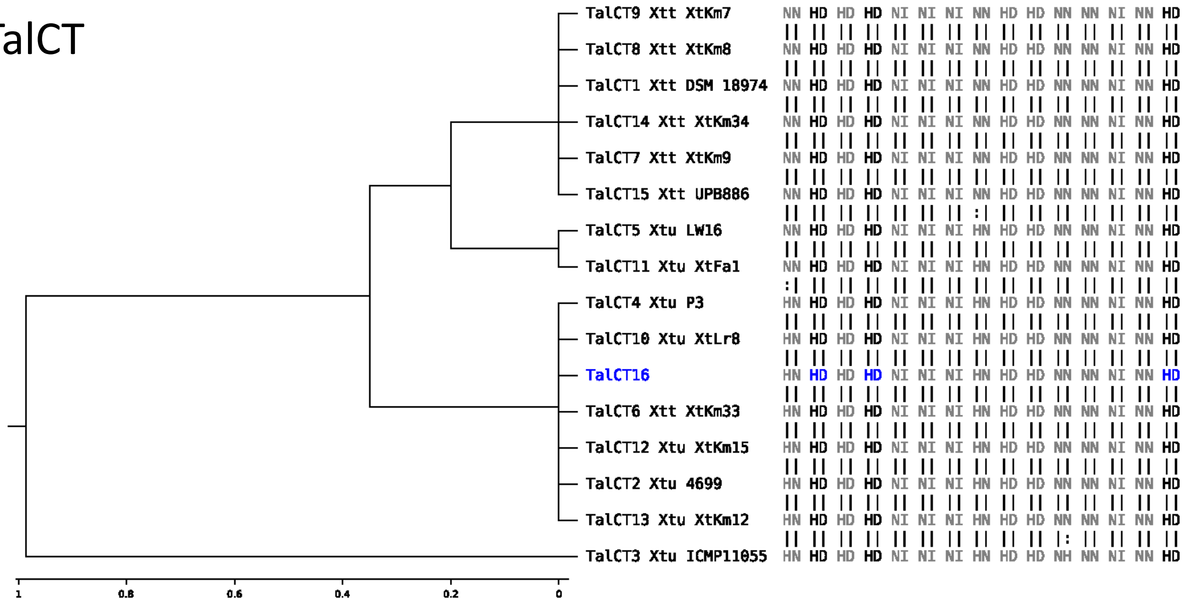

Supplemental Figure S5. TALE classes in *X. translucens* pv. undulosa. TALE classes assigned by AnnoTALE. Xtu CO237 TALEs are highlighted in blue in each tree class (continued from previous page).

A

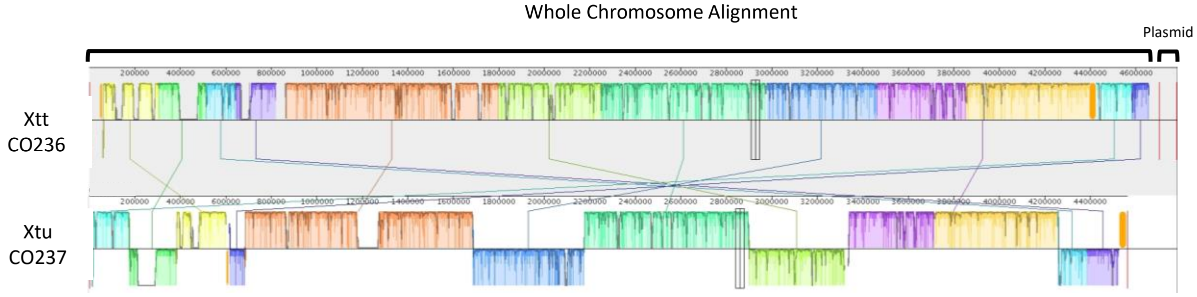

B

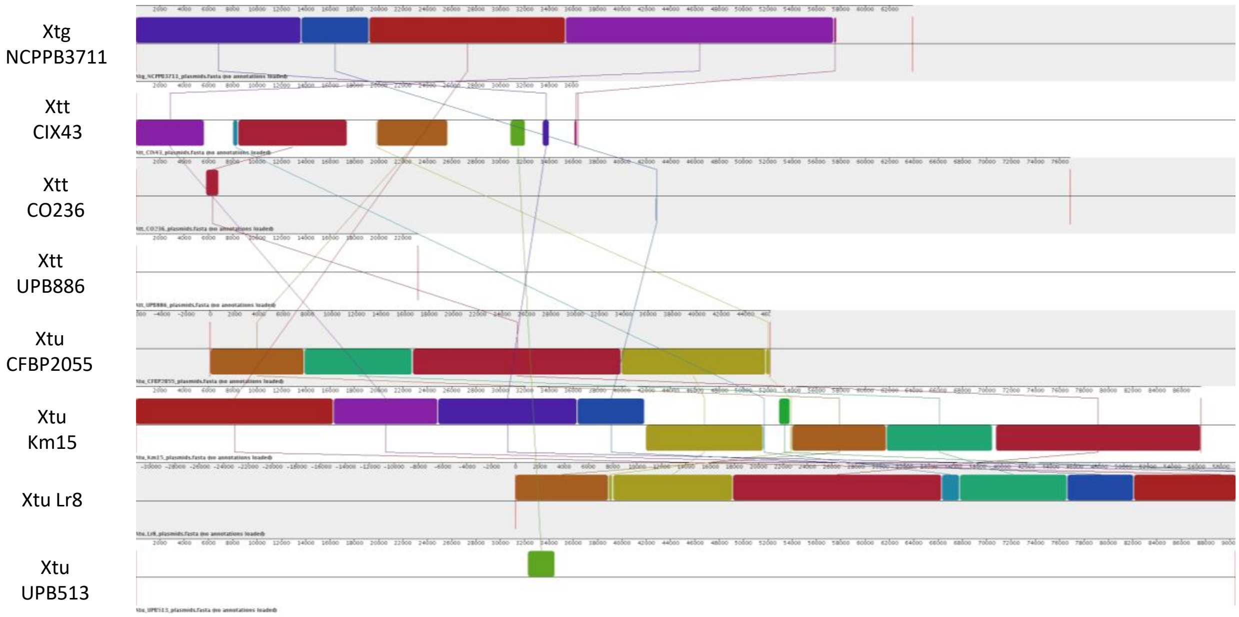

Supplemental Figure S6. Whole chromosome alignment between Xtt CO236 and Xtu CO237. **A.** Mauve alignment with whole-genome sequences of two Colorado strains show highly conserved regions in the main chromosome, but not the plasmid (marked at the end of Xtt CO236 sequence map). **B.** Mauve alignment with chromosomes from Xtt CO236 and all plasmids from *X. translucens* genomes available. There is high variability across plasmids between the Xt strains. Only an approximately 1kb fragment shares similarity between Xtt CIX43, Xtu CFPB2055, Xtu Km15, and Xtu Lr8 plasmids. Red lines denote beginning and end of contigs.
